# A pulmonary artery catheter-based treatment algorithm changes therapeutic behaviour in septic patients

**DOI:** 10.1186/cc9465

**Published:** 2011-03-11

**Authors:** C Bethlehem, FM Groenwold, MA Kuiper, M Koopmans, EC Boerma

**Affiliations:** 1 Medical Centre Leeuwarden, the Netherlands; 2 University Medical Centre Groningen, the Netherlands

## Introduction

For years the role of the pulmonary artery catheter (PAC) in ICU patients has been a topic of discussion. The use of PAC itself is not associated with improved outcome, and might contribute to increased morbidity [1]. However, the influence of a therapeutic strategy, based on dynamic PAC-derived variables, has never been investigated. The aim of this study is to evaluate whether such PAC-based strategy influences therapeutic behaviour in septic patients.

## Methods

We performed a single-centre retrospective case-control study in a 22-bed mixed ICU. Seventy patients with severe sepsis or septic shock, treated after introduction of a strict PAC-based resuscitation protocol, were compared with 70 matched controls, treated at the discretion of the attending physician. Continuous PAC measurements (Vigilance^®^) were started within 4 hours of admission. In short, the treatment algorithm only allowed infusion of fluids in cases of a 10% rise in left ventricular stroke volume; administration of dopamine was titrated on cardiac index in combination with central venous oxygen saturation. Norepinephrine was administered in cases of persistent hypotension despite the first two steps [2]. Primary outcomes were cumulative fluid balance and maximum dose of dopamine and norepinephrine in the first 24 hours. Statistical comparison between groups was performed with applicable tests; data are expressed as median (IQR).

## Results

At ICU admission there were no differences in severity of disease or predicted mortality using the APACHE IV model. The cumulative fluid balance in the first 24 hours was significantly higher in the PAC group, in comparison with controls (6.0 (4.3 to 7.5) l vs. 3.6 (1.8 to 5.0) l, *P *= 0.00). However, after 7 days cumulative fluid balance was significantly lower in the PAC group (7.5 (4.6 to 13.1) l vs. 13.0 (6.7 to 17.7) l, *P *= 0.002). Maximum dose of norepinephrine in the first 24 hours was significantly higher in the PAC group (0.12 (0.03 to 0.19) μg/kg/minute vs. 0.02 (0 to 0.07) μg/kg/minute, *P *= 0.00). No difference in use of dopamine was found. There was a significant difference in days on mechanical ventilation in favour of the PAC group (7 (5.0 to 11.3) days vs. 10 (5.8 to 18.3) days, *P *= 0.01).

## Conclusions

A treatment strategy, based on dynamic PAC-derived parameters in septic patients, significantly alters fluid administration, use of norepinephrine and days on mechanical ventilation, in comparison with historic controls.
